# Sarcoidosis manifesting as hepatic and splenic nodules mimicking ovarian cancer metastases: A case report

**DOI:** 10.3892/ol.2015.3566

**Published:** 2015-08-04

**Authors:** YOSHIKUNI YONENAGA, FUMIKI KUSHIHATA, HITOSHI INOUE, JOTA WATANABE, TAIJI TOHYAMA, ATSURO SUGITA, YASUTSUGU TAKADA

**Affiliations:** 1 Department of Hepatobiliary-Pancreatic and Breast Surgery, Ehime University Graduate School of Medicine, Toon, Ehime 791-0295, Japan; 2 Department of Surgery, Nagahama City Hospital, Nagahama, Shiga 526-8580, Japan; 3 Pathology Division, Ehime University Hospital, Toon, Ehime 791-0295, Japan

**Keywords:** sarcoidosis, hepatic involvement, splenic involvement, ovarian cancer metastasis, laparoscopic surgery

## Abstract

The current study presents a case of sarcoidosis manifesting as hepatic and splenic nodules, which was difficult to differentiate from ovarian cancer metastases. A 24-year-old female, who was previously diagnosed with right ovarian cancer and underwent surgery at the age of 21, was found to have two nodules in the spleen revealed by contrast-enhanced computed tomography (CT). ^18^F-fluorodeoxyglucose positron emission tomography/CT revealed two abnormal high uptake lesions in the spleen and one abnormal high uptake lesion in the liver. Under a diagnosis of hepatic and splenic metastases from right ovarian cancer, a laparoscopic splenectomy and partial hepatectomy were performed. Histopathological examination showed that a large number of non-caseating epithelioid cell granulomas formed these nodules, which was compatible with sarcoidosis. This case indicates that it is difficult to distinguish sarcoidosis from metastatic disease even using the latest modalities, and that laparoscopic surgery is a minimally invasive and useful tool for forming a differential diagnosis.

## Introduction

Sarcoidosis is a systemic granulomatous disorder of unknown etiology, with pulmonary findings in >90% of patients (1). The annual incidence rate of sarcoidosis in Japan is 1.01 cases per 100,000 individuals, and the age-specific incidence rates in females demonstrate a biphasic pattern with the first incidence peak in 25–39 year-old patients and the second incidence peak in patients in the fifth and sixth decades of life (2). This second peak is approximately twice the size of the first. Furthermore, in males the incidence rate peaks in 20–34 year-old patients. It has also been found that patients exhibiting abnormalities in the eye, skin and cardiac laboratory findings account for 54.8, 35.4 and 23.0% of cases, respectively. The outcome of sarcoidosis is classified into two groups: Spontaneous regression (self-limited disease) or progression of extensive fibrotic lesions as a postgranulomatous fibrosis (3). Patient prognosis varies depending on patient characteristics and clinical manifestations, such as age, gender, ethnicity, chest stage (4) and the number of extrapulmonary involvements. Respiratory failure, or central nervous system or myocardial involvement are the most common causes of mortality in patients with sarcoidosis (3). While extrapulmonary involvement is common, with involvement of any organ, but particularly the lymph nodes, skin, eyes, heart and central nervous system (1,2), it is rare to find extrapulmonary disease in isolation. The current study presents a case of exclusive extrapulmonary sarcoidosis with asymptomatic hepatic and splenic manifestations, but without signs in other organs. Written informed consent was obtained from the patient for inclusion in the present study.

## Case report

In March 2007, a 21-year-old female underwent a right salpingo-oophorectomy, partial omentectomy and appendectomy due to right ovarian cancer at the Department of Obstetrics and Gynecology, Ehime University Hospital (Toon, Japan). Pathological examination of the tumor revealed multiple mucinous cysts, lined by mucinous tumor cells. Although adenomatous and borderline malignant regions were identified in the tumor, partial areas of carcinoma were also present, which proliferated and invaded the stroma. Thus, the ovarian tumor was diagnosed as mucinous cystadenocarcinoma. After performing adjuvant chemotherapy with three 28-day cycles of carboplatin (2 AUC; days 1, 8 and 15) and paclitaxel (60 mg/m^2^; days 1, 8 and 15), follow-up care was conducted on an outpatient basis every 3 months.

**Figure 1.**
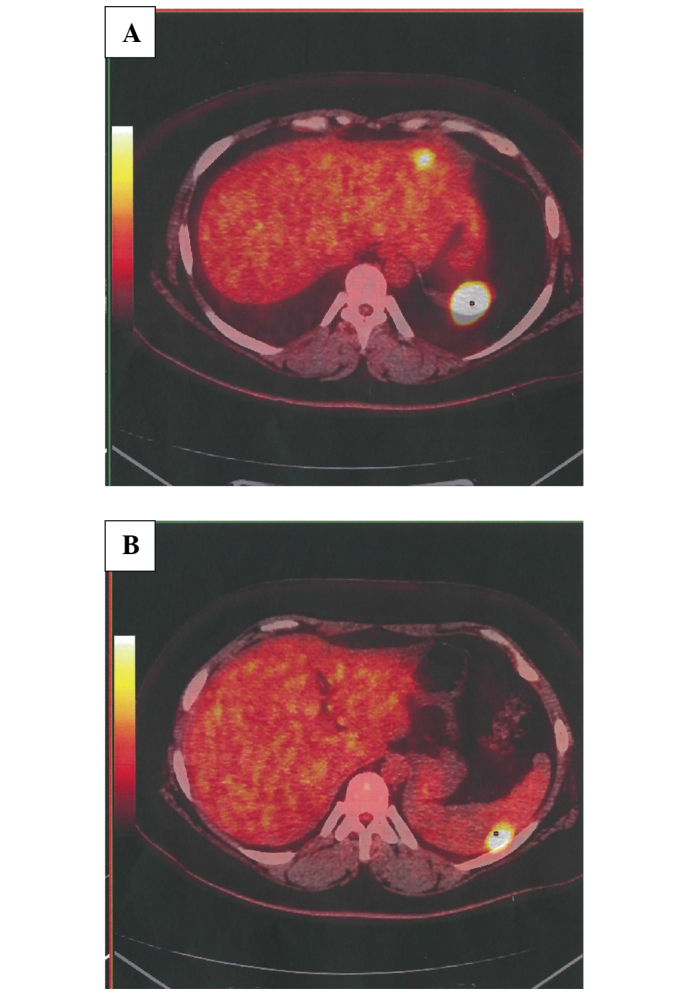
^18^F-fluorodeoxyglucose-positron emission tomography/computed tomography showing (A) a strong uptake lesion in the lateral segment of the liver, with a maximum standardized uptake value (SUV_max_) of 14.5 and (A and B) two strong uptake lesions in the spleen with a SUV_max_ of 21.0 and 18.2, respectively.

At 2 years and 6 months post-surgery, follow-up contrast-enhanced computed tomography (CT) showed two low density areas in the spleen. Thus, ^18^F-fluorodeoxyglucose (FDG)-positron emission tomography (PET)/CT was performed and revealed two abnormal high uptake lesions in the spleen, which were 2.5 and 1.0 cm in diameter, with a maximum standardized uptake value (SUV_max_) of 21.0 and 18.2, respectively. The PET/CT also showed an abnormal high uptake lesion in the lateral segment of the liver, which was 1.5 cm in diameter, with an SUV_max_ of 14.5 (Fig. 1).

**Figure 2.**
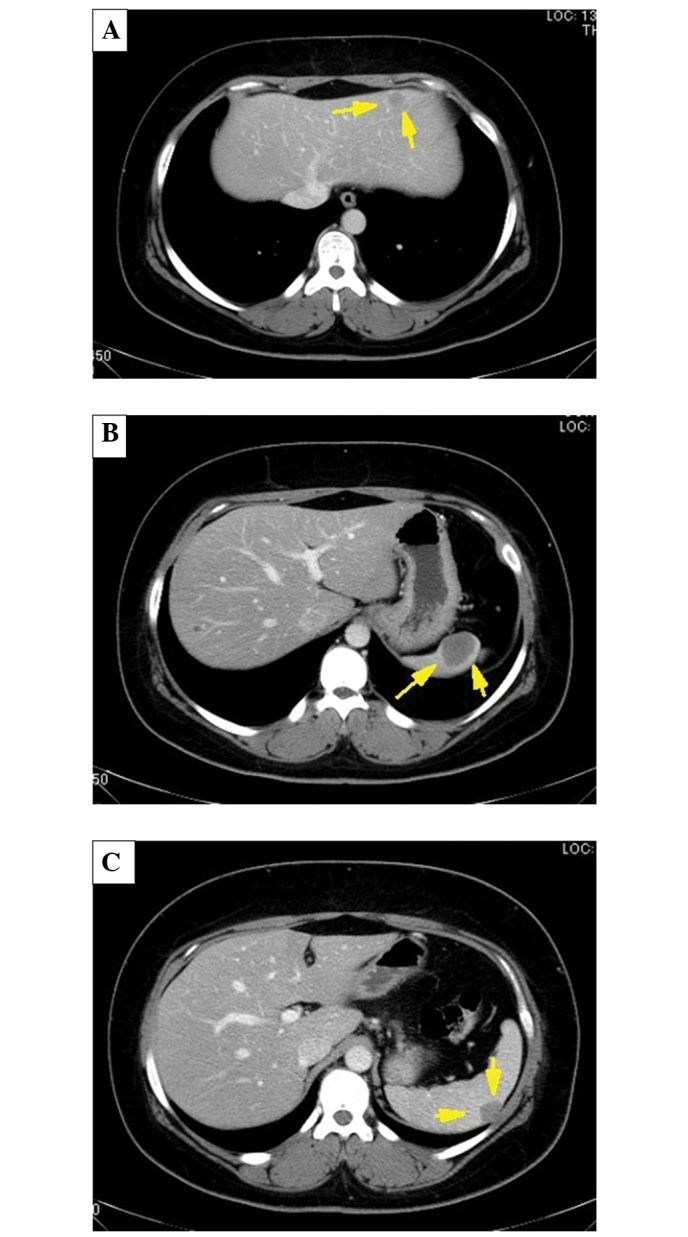
Contrast-enhanced computed tomography, which was performed again prior to surgery, revealing (A) a low-attenuation nodule (arrows) in the lateral segment of the liver and (B and C) two low-attenuation nodules (arrows) in the spleen.

**Figure 3.**
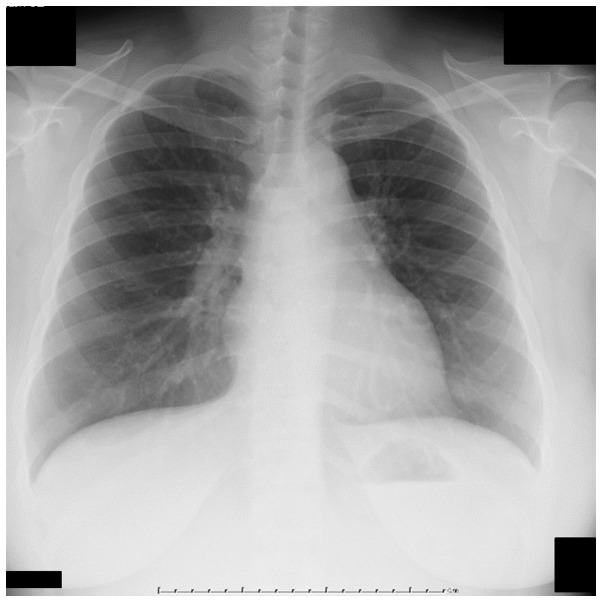
Chest roentgenogram showing no lung lesions or swollen hilar lymph nodes.

In October 2009, the patient was referred to the Department of Hepatobiliary-Pancreatic and Breast Surgery of Ehime University Hospital due to a working diagnosis of splenic and hepatic metastases from right ovarian cancer. A splenectomy and partial hepatectomy were recommended and the patient was admitted. Contrast-enhanced CT was performed again prior to admission and showed a low-attenuation nodule in the lateral segment of the liver, in addition to two low-attenuation nodules in the spleen (Fig. 2). Hepatomegaly and splenomegaly were not indicated on the radiology report. Abnormal lesions in the lung and bilateral hilar lymphadenopathy were also not indicated. At the time of admission, the patient was 162.3 cm tall and weighed 93.3 kg. The body mass index was 35.4 and the patient exhibited no swelling of the superficial lymph nodes. Routine blood analysis showed that the complete blood count, and test results for liver function, renal function and electrolytes, including serum calcium, were within normal limits, with the exception of a slightly elevated level of C-reactive protein (1.20 mg/dl; normal range, 0.00–0.20 mg/dl) (Table I). The serum levels of carcinoembryonic antigen, cancer antigen (CA)19-9, CA125 and α-fetoprotein were normal on admission, although the serum levels of CA19-9 (45 U/ml; normal range, <37 U/ml) and CA125 (56.8 U/ml; normal range, <35 U/ml) had been elevated prior to the previous surgery for right ovarian cancer. Electrocardiogram and chest X-ray results were normal (Fig. 3).

**Figure 4.**
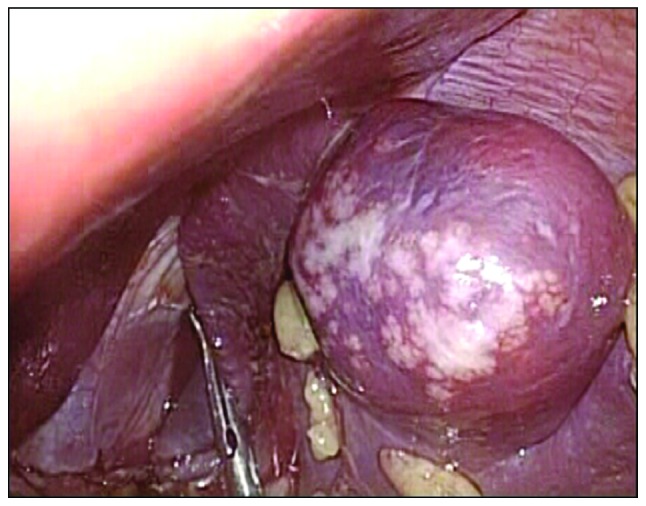
Laparoscopy image showing a tumor-like mass on the spleen.

**Figure 5.**
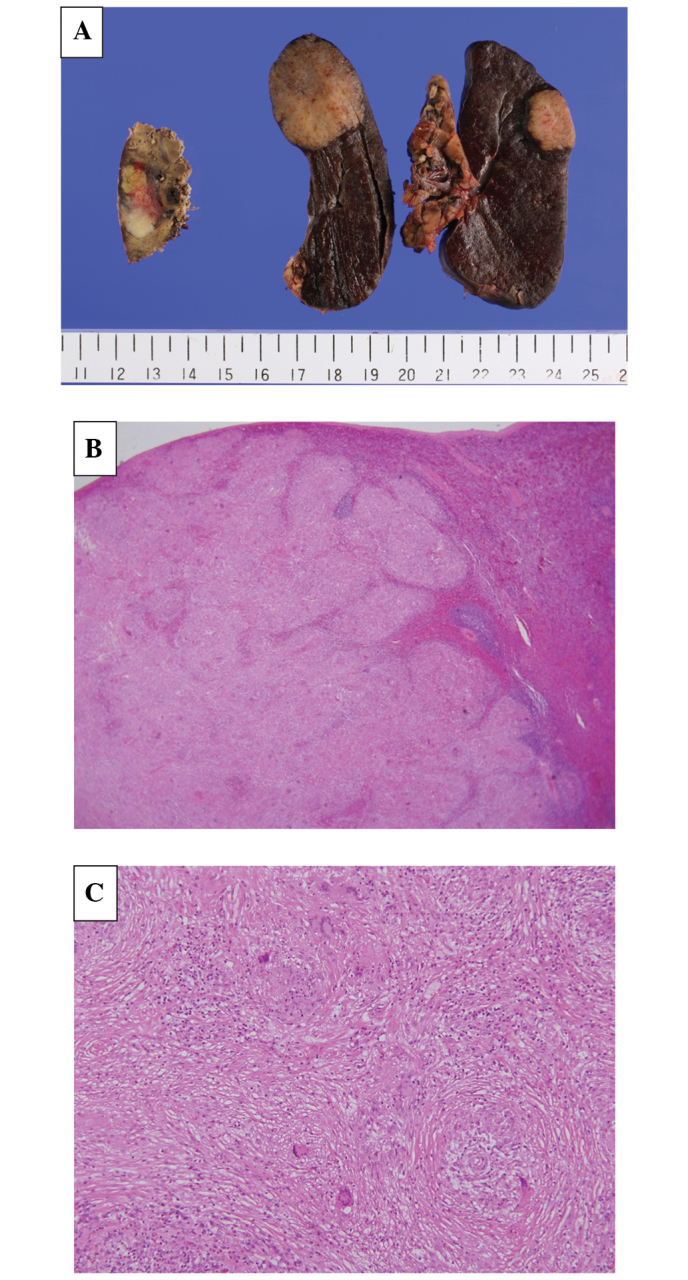
(A) Macroscopic image showing two yellow-white, elastic-hard nodules in the removed spleen, weighing 269 g, and a yellow-white, elastic-hard nodule in the resected liver specimen, weighing 34 g. (B and C) Pathological study revealing that a large number of non-caseating epithelioid cell granulomas formed these nodules. Hematoxylin and eosin staining at (B) x40 and (C) x200 magnification.

Due to the fact that the main differential diagnosis was splenic and hepatic metastases from right ovarian cancer, a laparoscopic procedure was performed. At the beginning of the laparoscopic procedure, an exploration of the abdominal cavity by a gynecologist was performed, which confirmed no abnormalities in the uterus or the adnexa of uterus, and no ascites or peritoneal dissemination. A cytological sample of peritoneal washing in the pouch of Douglas was obtained, and the presence of no malignant cells in the sample was confirmed the following day. The laparoscopic splenectomy and partial hepatectomy were then performed. Tumors were observed on the surface of the spleen (Fig. 4). Although a tumor was not observed on the liver, a 1.5-cm low echoic mass in segment 3 (Couinaud classification) (5) of the liver was confirmed by intraoperative ultrasonography. The whole spleen was removed after a 12-mm median incision for a port was widened by 4 cm. The weight of the spleen was 269 g. The weight of the resected liver specimen was 34 g. Macroscopically, yellow-whitish and elastic-hard nodules were found in the specimens on the sectional views (Fig. 5A). Histopathological examination showed that these nodules contained no malignant cells and that a large number of non-caseating epithelioid cell granulomas formed these nodules (Fig. 5B, C). Acid-fast bacillus was not detected using Ziel-Nielsen staining. Fungus was also not detected using periodic acid-Schiff staining and Grocott staining. Thus, the final diagnosis was of extrapulmonary sarcoidosis of the liver and spleen. Further post-operative laboratory studies showed no angiotensin-converting enzyme (ACE; 11.4 IU//l; normal range, 8.3–21.4 IU/l) or lysozyme (6.5 µg/ml; normal range, 5.0–10.2 µg/ml) abnormalities.

The patient recovered well with no further treatment, such as steroid therapy, and neither recurrence of sarcoidosis in any other organs nor recurrence and metastasis of right ovarian cancer have been observed for 2 years and 5 months since the surgery. At 5 years and 6 months post-surgery the patient remained alive.

## Discussion

Sarcoidosis is a disease of unknown cause, characterized by the presence of non-caseating granulomous lesions in multiple organs. Autopsy studies have demonstrated liver and spleen involvement in 44.6 and 41.4% of patients, respectively (6). However, autopsy studies in general tend to place emphasis on patients with more advanced disease states. Clinically, Morimoto *et al* reported that the rate of hepatic involvement was 5.6% among newly biopsy-proven sarcoidosis patients in Japan (2). The rate of splenic involvement was not documented in the study. A Case Control Etiologic Study of Sarcoidosis (ACCESS) reported that the rates of hepatic and splenic involvement were 11.5 and 6.7%, respectively, among newly biopsy-confirmed sarcoidosis patients from several geographical regions of the United States (1).

The initial presentation of sarcoidosis with hepatic and splenic involvement is rare. In the present study, the pre-operative examinations, including X-ray, CT and PET/CT, did not show any other abnormalities, particularly pulmonary involvement. Hepatomegaly is the most commonly noted radiographic finding of hepatic sarcoidosis (7). In the majority of patients, the liver appears homogeneous. Focal nodules of the liver are also observed in patients with hepatic sarcoidosis. Pathologically, these nodules are believed to represent the coalescence of small granulomas into macroscopically visible lesions (8). In the present case, histopathological examination also showed that non-caseating epithelioid cell granulomas formed the hepatic and splenic nodules. Such nodules typically show a diffuse distribution and cannot be counted (7), with sizes ranging from a few millimeters up to several centimeters (9,10). In addition, Warshauer *et al* reported that hepatomegaly, splenic nodules and abdominal lymphadenopathy are frequently associated with the presence of hepatic nodules (9). Thus, the present case was extremely rare, as only one nodule was observed in the liver, and hepatomegaly and abdominal lymphadenopathy were not observed. The most common radiographical finding of splenic sarcoidosis is splenomegaly (7). Hypodense splenic nodules may also be observed in patients with sarcoidosis; the majority are between 0.1 and 3.0 cm in diameter, with a mean of ~1.0 cm (9,10). Although splenomegaly is common, Warshuer *et al* reported that 17% of patients with nodules were found to have normal-sized spleens (9). Lesions usually show a diffuse distribution and are innumerable. However, in the present case, only two nodules were observed in the spleen.

Hepatic and splenic involvement is usually asymptomatic, or hepatic involvement can be associated with a liver function abnormality (11). However, the severity of hepatic involvement varies from asymptomatic to portal hypertension and cirrhosis, which can require liver transplantation (11). In certain cases, sarcoidosis was diagnosed only after pathological examination of explanted livers (11). Notably, the recurrence of sarcoidosis on liver grafts has also been reported (11,12). Thus, Kennedy *et al* suggested that hepatic sarcoidosis can be a serious and rapidly progressive disease (11). In the present case, the patient did not suffer a recurrence of sarcoidosis in the remnant liver during the follow-up.

Splenic metastasis is uncommon (13), but certain studies have reported splenic parenchymal metastasis from ovarian cancer (14,15). PET/CT is an accurate and useful tool for diagnosing ovarian cancer recurrence, i.e., lymph node metastases, peritoneal implants and distant sites of metastasis, including the liver and spleen (16). Meanwhile, increased FDG accumulation has also been reported in sarcoidosis (17). The emergence of novel modalities, such as PET/CT, can increase the chances of detecting incidental sarcoidosis lesions (18). In the present case, contrast-enhanced CT, which was performed 3 weeks prior to the PET/CT procedure, detected two low-density nodules in the spleen, but a hepatic nodule was not observed by two radiologists. However, such sensitive activity of PET/CT can mimic neoplastic or infectious disease in a patient in whom sarcoidosis is asymptomatic and undiagnosed (7).

In the present case, magnetic resonance imaging (MRI) was not performed pre-operatively. MRI may show foci of hypointensity on T2-weighted images in the liver, whereas neoplastic and other diseases tend to produce hyperintense signals (19). If MRI had been performed, it may have differentiated hepatic sarcoidosis from liver metastasis in this case.

Additionally, there was no evidence of hypercalcemia, nor elevated levels of serum ACE and lysozyme in the present case. Epithelioid cells are believed to produce serum ACE within sarcoid granulomas. Warshauer *et al* indicated that an elevated ACE level may be useful in the diagnosis of nodular hepatosplenic sarcoidosis (9). The study reported an elevated ACE level in 10 out of 11 patients studied, and the single patient with unaffected levels had been receiving prednisone treatment. However, in the present case, the serum level of ACE, which was measured at 27 days post-surgery, was normal. This normal level of ACE may be attributed to the resection of the nodules.

Following diagnosis, continual follow-up for systemic manifestations and complications associated with sarcoidosis is required. Steroids are an important base in the treatment of active sarcoidosis. In the present case, steroids were not used after surgery, and the only follow-up care was conducted on an outpatient basis. Although it has been reported that the lungs are involved in >90% of patients (1), pulmonary abnormalities did not subsequently appear in the 2 years and 5 months post-surgery.

Compared with previous studies of hepatosplenic sarcoidosis (7–9,20), the present case was rare due to the small number of nodules, the presence of two nodules in the spleen and one nodule in the liver, and the lack of lung involvement. These unusual and less frequent presentations are difficult to diagnose in the absence of clinical suspicion of sarcoidosis.

In conclusion, the aforementioned unusual findings of hepatic and splenic sarcoidosis in the present case made it difficult to differentiate between sarcoidosis and metastases from ovarian cancer. This case suggests that it is difficult to distinguish sarcoidosis from metastatic disease even using the latest modalities, and that laparoscopic surgery is a minimally invasive and useful tool for forming a differential diagnosis.

## Figures and Tables

**Table I.** Laboratory findings.

Factor	Value
White blood cells, x10^3^/µl	5.3
Red blood cells, x10^6^/µl	4.88
Hemoglobin, g/dl	13.8
Hematocrit, %	41.9
Platelets, x10^5^/µl	2.7
C-reactive protein, mg/dl	1.20
Glutamate-oxaloacetate transaminase, IU/l	25
Glutamate-pyruvate transaminase, IU/l	39
Total protein, g/dl	7.2
Albumin, g/dl	4.0
Total bilirubin, mg/dl	0.3
Creatinine, mg/dl	0.7
Blood urea nitrogen, mg/dl	15
Blood sugar, mg/dl	79
Amylase, IU/l	67
Sodium, mEq/l	139
Potassium, mEq/l	3.8
Chloride, mEq/l	106
Calcium, mg/dl	9.1
Carcinoembryonic antigen, ng/ml	1.1
Cancer antigen 19-9, U/ml	12
Cancer antigen 125, U/ml	6.7
α-fetoprotein, ng/ml	2.1
